# TTK (threonine tyrosine kinase) regulates the malignant behaviors of cancer cells and is regulated by microRNA-582-5p in ovarian cancer

**DOI:** 10.1080/21655979.2021.1968778

**Published:** 2021-09-13

**Authors:** Na Lu, Lixin Ren

**Affiliations:** a Department of Gynecology, Shanxi Provincial Cancer Hospital, Taiyuan, Shanxi China; b Department of General Surgery, Shanxi Provincial Cancer Hospital, Taiyuan, Shanxi China

**Keywords:** MiR-582-5p, TTK, ovarian cancer

## Abstract

There is growing evidence that threonine tyrosine kinase (TTK) dysregulation is linked to the progression of multiple malignancies. Nonetheless, the role of TTK in ovarian cancer (OC) remains unclear. The GEO2R method was employed to screen out the mRNAs that were abnormally expressed between OC tissues and normal ovarian tissues using three datasets from the Gene Expression Omnibus (GEO) database: GSE14407, GSE18520, and GSE36668. Moreover, the Kaplan–Meier plotter was utilized to investigate the association between TTK expression and OC patients’ prognosis. Furthermore, quantitative real-time PCR (qRT-PCR) was applied to examine miR-582-5p expression and TTK mRNA expression in OC tissues and cells. Additionally, immunohistochemistry (IHC) experiment and Western blot were executed to examine TTK protein expression in OC tissues and cells, respectively. In addition, Cell Counting Kit-8 (CCK-8), transwell, and flow-cytometry experiments were performed to examine the multiplication, migration, and apoptosis of OC cells, respectively. In addition, dual-luciferase reporter gene tests were executed to validate the targeting relationship between miR-582-5p and TTK. We demonstrated that TTK expression was up-regulated in OC tissues and cells, and its overexpression was found to be associated with an adverse prognosis in OC patients. TTK overexpression enhanced OC cell multiplication and migration, and repressed apoptosis. Mechanistically, TTK was a downstream target of miR-582-5p. Furthermore, miR-582-5p overexpression impeded OC cell multiplication and migration, while TTK overexpression reversed this phenomenon. These data suggest that miR-582-5p and TTK are promising targets for OC diagnosis and therapy.

## Introduction

Ovarian cancer (OC) is a common malignancy for women[1]. Over 70% of OC patients are diagnosed as advanced Federation International of Gynecology and Obstetrics (FIGO) stage (III or IV) [2–5]. Deciphering the mechanism of OC progression is essential for early diagnosis as well as personalized treatment.

TTK (threonine tyrosine kinase) protein kinase, also known as monopolar spindle 1 (MPS1), is a crucial modulator of the spindle assembly checkpoint, which is responsible for maintaining genomic integrity[6]. TTK has shown the potential as a therapeutic target for tumors. TTK expression is associated with the malignancy of triple-negative breast cancer cells[7]. Another study reports that TTK is a prognostic biomarker for endometrial cancer[8]. Targeting TTK is a promising strategy for cancer treatment, and several TTK inhibitors are being tested in pre-clinical studies and clinical trials[9]. For example, TTK inhibitor AZ3146 induces cell cycle arrest of liver cancer cells, and enhances DNA damage, apoptosis, and necrosis induced by radiation[10]. TTK has been reported to regulate the progression of several cancer such as hepatocellular carcinoma, lung cancer, and glioma [11–13]. Nonetheless, the function and mechanism of TTK in OC are blurred.

MicroRNAs (miRNAs) are short non-coding RNAs with 20–22 nucleotides that bind with the 3ʹ-UTR of mRNA and cause mRNA degradation or translation inhibition[2]. Reportedly, miRNAs regulate more than 30% of human genes, with a single miRNA controlling hundreds of target genes[14]. MiRNAs modulate a wide range of biological processes, including cell growth, differentiation, apoptosis, migration, and invasion, as well as play a crucial role in tumorigenesis [15,16]. A growing body of evidence suggests that abnormal miRNA expression is related to the development of OC. For instance, miR-205 overexpression in circulating exosomes is linked to OC metastasis, and it is abundant in the serum of OC patients; miR-205 induces angiogenesis and accelerates tumor growth via PTEN-AKT pathway[17]. MiR-582-5p is reported to be a tumor suppressor in multiple cancers[18], while its role in OC remains unclear.

In this study, we hypothesized that TTK was associated the progression of OC. With bioinformatics analysis and *in vitro* experiments, the expression characteristics, biological function, and regulatory mechanism of TTK in OC were investigated. Here we report that TTK, a target gene of miR-582-5p, is up-regulated in OC, and regulates the malignant phenotypes of OC cells.

## Materials and methods

### Subjects and specimens

This study enrolled 32 participants who were diagnosed with OC and had undergone surgical resection at Shanxi Provincial Cancer Hospital. The OC tissues and paracancerous normal tissues of the subjects were taken and immediately frozen in liquid nitrogen. This research was endorsed by the Ethics Committee of Shanxi Provincial Cancer Hospital and written informed consent was available from the subjects.

### Microarray data

Gene Expression Omnibus (GEO) database was utilized to obtain the microarray data. Three datasets (GSE14407, GSE18520, and GSE36668) were downloaded and analyzed, which met the following criterion: containing the gene expression data from both OC tissues and normal (or adjacent) samples. GEO2R tool was adopted to filter the differently expressed genes between normal tissue samples and OC samples. The screening thresholds for genes were set at *P < *0.05 and │log2 (Fold Change)│ > 2.

### Cell culture

Cell culture was performed as previously described[19]. Human OC cell lines (SKOV-3, OVCAR-3, H8910) were procured from American Type Culture Collection (ATCC, Rockville, MD, USA). Normal ovarian epithelial cell line HOSE was available from the Cell Bank of the Chinese Academy of Sciences (Shanghai, China). At 37°C with 5% CO_2_, all cells were cultured in Dulbecco’s modification of Eagle’s medium (DMEM) (Invitrogen, Carlsbad, CA, USA) with 10% heat-inactivated fetal bovine serum (Gibco, Grand Island, NY, USA), 100 U/ml penicillin and 100 μg/ml streptomycin (Gibco, Grand Island, NY, USA). pcDNA3.1-TTK, empty vector, TTK siRNA (si-TTK#1/si-TTK#2), scramble siRNA, miR-582-5p mimics, miR-582-5p inhibitors and negative control (miR-NC) were procured from Invitrogen (Carlsbad, CA, USA). SKOV-3 and OVCAR-3 cells were transfected with the oligonucleotides or plasmids with Lipofectamine® 3000 (Invitrogen, Carlsbad, CA, USA).

### Quantitative real-time PCR

RNA extraction was performed with TRIzol method[20]. Briefly, tissue samples were fully homogenized with an electric homogenizer, then added with TRIzol reagent (Yeasen Biotech, Shanghai, China), and incubated for 5 min at room temperature. Next, chloroform was added, and fully mixed, and then incubated for 2 min at room temperature. After centrifugation (12,000 rpb, 4°C, 5 min), the precipitation was discarded. Next, isopropanol was added in the supernatant, and incubated for 10 min. Then the precipitation was collected, and dissolved in RNase-free water. For reverse transcription, a PrimeScript RT Master Mix Kit (Takara Bio, Tokyo, Japan) was used for TTK, and a TaqMan MicroRNA reverse transcription kit (Applied Biosystems, Foster City, CA) was used for miR-582-5p. Then, with the SYBR® Premix Ex Taq^TM^ II (Takara Bio, Tokyo, Japan), and cDNA, qRT-PCR was performed to detect the relative expression of TTK. To assess miR-582-5p expression, qRT-PCR was conducted using stem-loop primer SYBR Green qRT-PCR kit (Synbio Tech, Suzhou, China). 2^−ΔΔCt^ approach was used to measure the relative expressions of miR-582-5p and TTK using U6 and GAPDH as endogenous controls. The primer sequences are shown in Table 1.

**Table 1.** Primer sequences

Name	Primer sequences
miR-582-5p	Forward: 5ʹ-GCGGTTACAGTTGTTCAACC-3’
	Reverse: 5′-CTCAACTGGTGTCGTGGA-3′
TTK	Forward: 5′-CCGGAGTTAGCCCGAAAAGT-3′
	Reverse: 5′-AGGTATTGCTGCTTGGTGTCT-3′
U6	Forward: 5ʹ-GCTTCGGCAGCACATATACTAAAAT-3’
	Reverse: 5ʹ-CGCTTCAGAATTTGCGTGTCAT-3’
GAPDH	Forward: 5ʹ-TGGCACCCAGCACAATGAA-3’
	Reverse: 5ʹ-CTAAGTCATAGTCCGCCTAGAAGCA-3’

### Immunohistochemistry (IHC)

As previously described[21], briefly, the tissue blocks were sliced, and the sections were deparaffinized and rehydrated. Then the sections were heated for 30 min in 10 mM sodium citrate buffer (pH 6.0) at 95–100°C. Then the sections were blocked with 3% bull serum albumin for 1 h at room temperature. Then the sections were incubated with anti-TTK antibody (1: 100, Abcam, Cambridge, UK) at 4°C overnight, then incubated with secondary antibody (Beyotime, Shanghai, China) for 1 h at room temperature. Then, the sections were stained with diaminobenzidine and observed under a microscope. The color development was terminated after the color of the tissues turned yellow. Next, the staining intensity was scored by a pathologist.

#### Cell counting kit-8 (CCK-8) assay

Cell growth was monitored by CCK8 assay as previously reported[22]. Cells were plated in 96-well plates (2 × 10^3^cells/well) with 100 μL of medium per well, and then the cells were routinely cultured. 10 μL of CCK-8 solution (Beyotime, Shanghai, China) was supplemented to each well at 0 h, 24 h, 48 h, 72 h and 96 h, respectively. Then the cells were incubated at 37°C for 2 h, and the absorbance at 450 nm was examined with a microplate reader.

#### Transwell assay

Transwell assay was used to detect the migration of OC cells[23]. The OC cells of each group in logarithmic growth phase were trypsinized with 0.25% trypsin, and re-suspended with serum-free medium, with the cell concentration adjusted to 1 × 10^5^ cells/mL. 200 μL of cell suspension was supplemented to the top compartment of each Transwell chamber (BD Biosciences, San Jose, CA, USA), and 500 μL of medium containing 10% fetal bovine serum was supplemented to the bottom compartment, and then the cells were cultured for 24 h. Then, methanol was used to fix the cells on the lower surface of the membrane, and 0.1% crystal violet was used to dye them. In five fields of view under the microscope, the number of cells was counted, and the average was calculated.

#### Apoptosis experiment

The apoptosis experiment was performed according to the previously reported method[24]. An FITC-Annexin V and PI Apoptosis Detection kit (BD Biosciences, San Jose, CA, USA) was used. After being washed twice with pre-chilled phosphate buffer saline (PBS), OC cells (3 × 10^5^ cells per sample) were resuspended in 1 mL of binding buffer, and then 5 μL of FITC-annexin V staining solution was added, and incubated in the dark for 30 min. Following that, 5 μL of propidium iodide (PI) was added, and then the cells were incubated in the dark for 30 min. Then a flow cytometer (BD Biosciences, San Jose, CA, USA) was utilized to examine the apoptosis of the cells, and the data were analyzed using FlowJo (BD Biosciences, Franklin Lake, NJ, USA).

#### Western blot

Western blotting was performed as previously described[23]. The cells were lyzed for 10 min in RIPA lysis buffer (Beyotime, Shanghai, China), and after centrifugation, the supernatant of the lysate was used as the protein sample. Subsequently, a BCA Protein Assay Kit (Beyotime, Haimen, China) was utilized to determine protein concentration. Next, the proteins were dissolved by sodium dodecyl sulfate polyacrylamide gel electrophoresis (SDS-PAGE) and transferred to polyvinylidene fluoride (PVDF) membranes (Millipore, Billerica, MA, USA), which was then blocked with 5% skim milk for 1 h at room temperature. Next, primary antibodies anti-TTK (1:300, ab11108, Abcam, Cambridge, UK) and anti-GAPDH (1:2000, ab8245, Abcam, Cambridge, UK) were applied and the PVDF membranes were incubated at 4°C overnight. Thereafter, the membranes were incubated with the horseradish peroxidase-conjugated secondary antibody (1: 2000, Beyotime, Shanghai, China) for 50 min at room temperature. An electrochemiluminescence kit (Biosharp, Hefei, China) was utilized to develop the protein bands, and Amersham Imager 600 (GEHealthcare, Chicago, IL, USA) was used to detect the protein bands.

### Luciferase reporter experiment

Bioinformatics analysis was used to predict the presence of binding sequence between miR-582-5p and TTK 3ʹUTR, and the binding fragment of TTK 3ʹUTR to miR-582-5p was amplified using PCR. The amplification product was inserted into the empty luciferase reporter vector (Promega, Madison, WI, USA) to establish TTK 3ʹUTR wild-type (wt) plasmids, and the binding fragment was mutated using gene mutation technique to construct TTK 3ʹUTR mutant (mut) plasmids. The recombinant plasmids were co-transfected into HEK-293 T cells with miR-582-5p mimics or miR-NC, respectively. 48 h later, the cells were collected, and the luciferase activity of the cells was determined with a dual-luciferase reporter gene assay system (Promega, Madison, WI, USA)[25].

### Statistical analysis

All of the experiments were performed in triplicate. All the data were shown as mean ± standard deviation. GraphPad Prism 8 (GraphPad Software, Inc., La Jolla, CA, USA) was applied for graphing and statistical analysis. Kolmogorov–Smirnov test was used to examine the normality of the data. For normally distributed data, student’s *t*-test was executed for comparisons of the means of the data in two groups, and one-way ANOVA, with Tukey’s post-hoc test, was conducted for comparison of the means of the data in three or more groups. For skewed data, comparisons between the two groups were performed with Wilcoxon signed-rank test. Kaplan–Meier plots and log-rank test were employed to perform survival analysis. Pearson correlation coefficient was utilized to examine the correlations among gene expressions. A value of *P* < 0.05 signified statistical significance.

## Results

The present study was designed to investigate the expression characteristics, biological functions, and upstream mechanism of TTK in OC, with *in vitro* experiments.

### TTK overexpression is associated with the poor prognosis of OC patients

**Figure 1.**
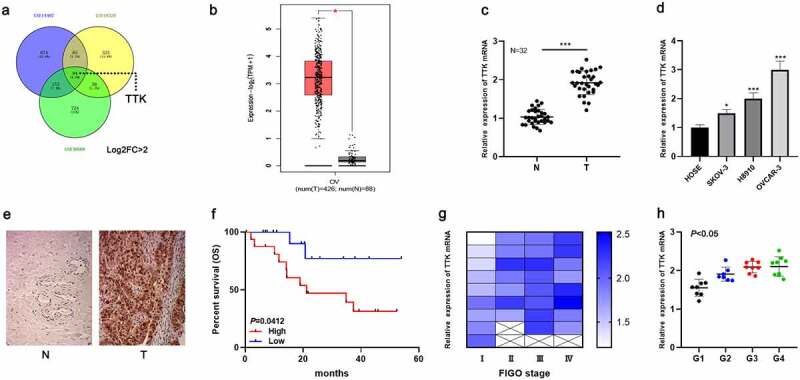
TTK overexpression is significantly up-regulated in OC, and is associated with the prognosis of OC patients

Three gene expression datasets (GSE14407, GSE18520, and GSE36668) were collected from the GEO database, and differentially expressed genes between normal ovarian tissues and OC tissues were screened out with GEO2R tool. A venn diagram was utilized to get the genes whose expression was up-regulated in all of the three datasets, and nine genes were obtained, including TTK (Figure 1a). Moreover, data from the Gene Expression Profiling Interactive Analysis 2 (GEPIA2) database revealed that TTK expression was remarkably higher in OC tissues than in normal ovarian tissues, which was consistent with the data of the GEO datasets (Figure 1b). Additionally, the results of qRT-PCR revealed that TTK expression was markedly augmented in OC tissues compared with the control tissues (Figure 1c). Also, TTK expression was remarkably augmented in OC cell lines (SKOV-3, OVCAR-3, H8910) relative to normal ovarian epithelial cell lines (HOSE) (Figure 1d). IHC staining revealed a higher positive rate of TTK in OC tissues (25/32, 78.1%) compared with normal tissues adjacent to cancer (6/32, 18.8%) (Figure 1e), suggesting that the expression level of TTK protein was also up-regulated in the tumorigenesis of OC. Importantly, high expression of TTK was associated with shorter survival time of the OC patients (figure 1f). Additionally, the expression level of TTK was positively associated with FIGO stage and differentiation grade of the OC patients (Figure 1g-h). These data, collectively, implied that TTK was overexpressed in OC, and its high expression indicated the unfavorable prognosis of the patients.

A. Venn diagrams illustrating the number of up-regulated differentially expressed genes (DEGs) in three gene expression datasets (GSE14407, GSE18520 and GSE36668). B. GEPIA2 database was used to analyze TTK expression in OC tissues (red), compared with normal ovarian tissues (gray). C. TTK expression in OC tissues and normal tissues was detected by qRT-PCR. D. TTK expression in OC cell lines (SKOV-3, OVCAR-3 and H8910) and normal ovarian epithelial cell lines (HOSE) was detected by qRT-PCR. E. IHC staining was employed to detect TTK protein expression in OC tissue and paracancerous tissue. F. Kaplan-Meier plots were utilized to analyze the relationship between TTK expression and overall survival of OC patients. G. The association between TTK expression and FIGO stage of OC patients. H. The association between TTK expression with differentiation grade of OC patients. * *P* < 0.05, and *** *P* < 0.001.

### TTK facilitates OC cell growth and migration and inhibits apoptosis

**Figure 2.**
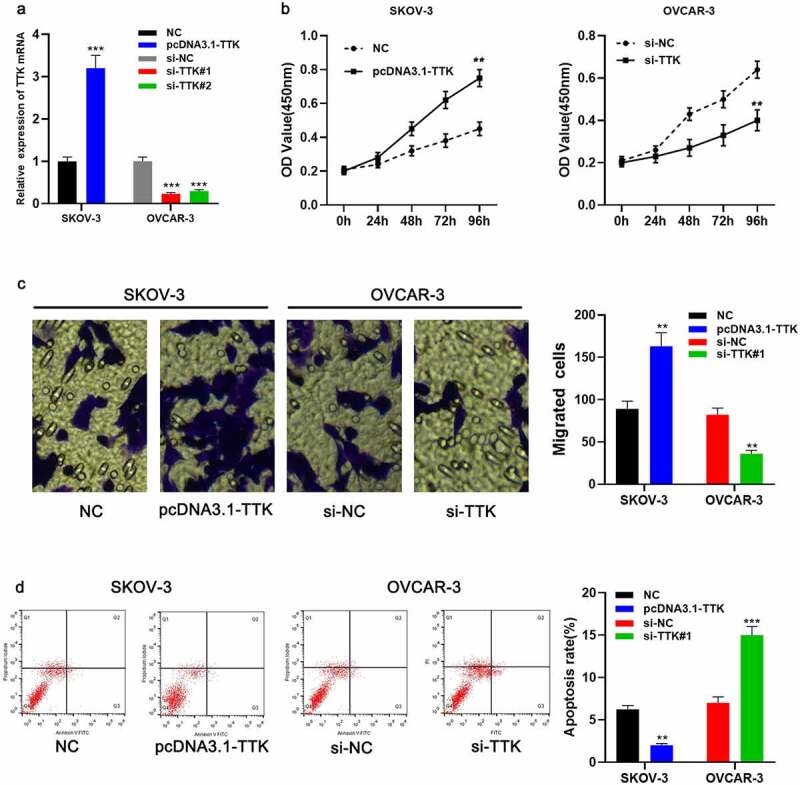
TTK enhances OC cell growth and migration and inhibits apoptosis

To decipher the function of TTK in OC progression, pcDNA3.1-TTK and si-TTK#1/si-TTK#2 were transfected into SKOV-3 and OVCAR-3 cells, respectively, and TTK overexpression and TTK knockdown cell models were constructed (Figure 2a). si-TTK#1 showed higher efficiency of TTK knockdown, so it was used for the subsequent experiments. The data of the CCK-8 experiment revealed that in TTK overexpression group, cell viability was markedly higher than that in the control group (Figure 2b). The data of the Transwell assay revealed that the migration of cells in TTK overexpression group was notably higher than that of the control group (Figure 2c). Furthermore, the cells’ apoptosis rate in TTK overexpression group was markedly lower than that in the control group (Figure 2d). In addition, knockdown of TTK resulted in reduced growth and migration of OVCAR-3 cells and increased apoptosis rate (Figure 2b-d). These results implied that TTK worked as an oncogenic factor in OC.

A. The pcDNA3.1-TTK and si-TTK#1/si-TTK#2 were transfected into SKOV-3 and OVCAR-3 cell lines, respectively, and the transfection efficiency was examined by qRT-PCR. B. CCK-8 assay was utilized to detect the growth of OC cells. C. Migration assay was executed to detect the migration of OC cells. D. Flow cytometry was performed to detect the apoptosis rate of transfected OC cells. *P < 0.05, **P < 0.01, ***P < 0.001.

### TTK is a downstream target of miR-582-5p

**Figure 3.**
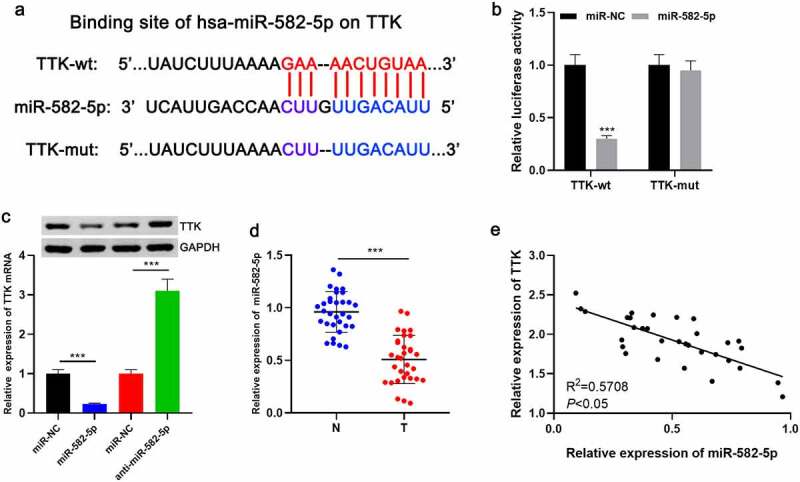
TTK is a downstream target of miR-582-5p

Next, miR-582-5p, was predicted by bioinformatics research to be a upstream regulator of TTK (Figure 3a). After the wild type and mutated type of luciferase reporters were constructed, dual-luciferase reporter gene experiment was performed, the result of which indicated that miR-582-5p repressed the luciferase activity of the wild-type reporter, but not that of the mutated reporter (Figure 3b). qRT-PCR and Western blot assays revealed that the transfection of miR-582-5p mimics significantly reduced TTK expression in OVCAR-3 cells compared with the control group; however, the transfection of miR-582-5p inhibitors significantly augmented TTK expression in SKOV-3 cells (Figure 3c). Furthermore, miR-582-5p expression in OC tissues was lower than that in paracancerous tissues, and miR-582-5p expression in OC tissues was negatively correlated with TTK expression (Figure 3d-e).

A. The binding site of miR-582-5p and TTK was predicted by bioinformatics analysis. B. Dual-luciferase reporter gene assay was used to validate the binding site between miR-582-5p and TTK. C. qRT-PCR and Western blot experiments were executed to detect the effects of miR-582-5p mimics and inhibitors on TTK expression. D. qRT-PCR was adopted to detect miR-582-5p expression in OC tissues. E. Pearson correlation coefficient analysis was applied to measure the correlation between miR-582-5p and TTK expression in OC tissues. *** *P* < 0.001.

### MiR-582-5p impedes the malignant phenotype of OC cells dependent on TTK

**Figure 4.**
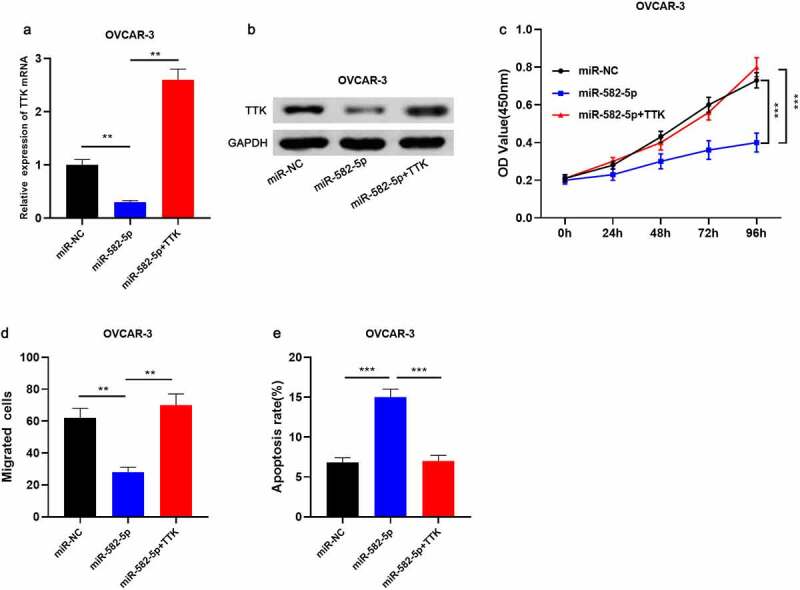
MiR-582-5p restrains the malignant phenotypes of OC cells via regulating TTK

To decipher the function of miR-582-5p/TTK axis in modulating OC cell growth, migration, and apoptosis, OVCAR-3 cells overexpressing miR-582-5p were co-transfected with pcDNA3.1-TTK, and qRT-PCR and Western blot were used to validate the transfection efficiency (Figure 4a-b). The results of CCK-8 and Transwell assays revealed that miR-582-5p overexpression significantly suppressed the viability and migration of OVCAR-3 cells, but TTK overexpression counteracted the impact of miR-582-5p (Figure 4c-d). Moreover, TTK overexpression reversed the apoptosis-promoting function of miR-582-5p on OVCAR-3 cells (Figure 4e). The findings suggested that miR-582-5p repressed the malignant phenotypes of OC cells via regulating TTK.

A-B. OVCAR-3 cells were transfected with miR-NC, miR-582-5p mimics, and miR-582-5pmimics + pcDNA3.1-TTK, respectively, and TTK expression in OVCAR-3 cells was detected by qRT-PCR and Western blot assays. C. CCK-8 assay was conducted to detect the growth of OC cells after the transfection. D. Transwell assay was performed to detect the migration of OC cells after the transfection. E. Flow cytometry was carried out to detect the apoptosis rate of transfected OC cells. * *P* < 0.05, ** *P* < 0.01, and *** *P* < 0.001.

## Discussion

TTK is a key component of the mitotic spindle assembly checkpoint (SAC) and it is an important regulator in mitosis [26,27]. Abnormality of TTK expression impairs SAC’s function, resulting in incorrect chromosomal segregation and aneuploidy, which can lead to apoptosis[27]. In gastric cancer, high expression of TTK is associated with poor prognosis of the patients, and depletion of TTK suppresses the proliferation and induces the apoptosis of gastric cancer cells via modulating Akt-mTOR signaling[28]. In colonic cancer, TTK, as an oncogene, promotes the proliferation of cancer cells via PKCα/ERK1/2 and PI3K/Akt pathways[29]. TTK expression is also linked to prostate cancer progression, and knockdown of TTK represses the malignancy of prostate cancer cells[27]. To the best of our knowledge, there was no study on the role of TTK in OC. In this work, TTK is revealed to be overexpressed in OC tissues and cells, and its overexpression is associated with unfavorable pathological characteristics and poor prognosis of the patients. These data suggest that TTK may be a prognostic biomarker for OC. This work also confirms that TTK overexpression enhances the proliferation and migration of OC cells and restrains apoptosis, whereas knockdown of TTK impedes the proliferation and migration of OC cells and induces apoptosis. Considering many TTK inhibitors are regarded as promising anti-cancer drugs[30], targeting TTK may be a novel strategy to treat OC, especially for the patients with advanced disease.

MiRNAs are short and highly conserved non-coding RNAs. These molecules modulate multiple biological processes via acting as a negative regulator of gene expression [31,32]. In cancer biology, the dysregulation of miRNAs contributes to tumorigenesis. It is reported that miR-182 inhibits the invasive phenotype of esophageal squamous cell cancer cells by regulating MYC proto-oncogene[33]. Another study reports that miR-936 inhibits glioma progression by regulating ErbB2 receptor tyrosine kinase 4[34]. MiR-582-5p is reported to be aberrantly expressed in diverse human cancers, including osteosarcoma, bladder cancer, non-small cell lung cancer (NSCLC), and endometrial cancer [35–38]. Specifically, miR-582-5p restrains the multiplication and invasion of osteosarcoma cells by targeting neuro-oncological ventral antigen 1[35]. MiR-582-5p blocks NSCLC metastasis by targeting mitogen-activated protein kinase kinase kinase 2, and it shows the potential to be an independent prognostic biomarker for patients with NSCLC[37]. In this work, we demonstrate that miR-582-5p is lowly expressed in OC tissues and cell lines, and TTK is validated as a target gene of miR-582-5p, and miR-582-5p can repress the malignant phenotypes of OC cells via repressing TTK. For the first time, we report the expression characteristics and function of miR-582-5p in OC, and our data suggest miR-582-5p/TTK axis is a novel mechanism of OC progression.

There are several limitations in the present work. First of all, only cell models were used to investigate the biological function of TTK in OC, and animal models are needed to validate our findings in the future. Additionally, the downstream mechanism by which TTK participates in OC progression is still obscure. Last but not least, other miRNAs that potentially target TTK remain to be screened out in the following work.

## Conclusion

Our study report that TTK is an novel oncogene in OC. Its expression level is up-regulated in OC tissues and cell lines, and its high expression implies worse prognosis of the patients. Also, TTK promotes the proliferation and migration, but represses the apoptosis of OC cells. MiR-582-5p is validated as an upstream regulator of TTK, and it represses the malignant phenotypes of OC cells via repressing TTK. Collectively, our study suggests that TTK and miR-582-5p are potential biomarker and therapy target for OC.

## Research highlights

- TTK is associated with the poor prognosis of patients with ovarian cancer.
- TTK is identified as a target gene of miR-582-5p.
- MiR-582-5p regulates the progression of ovarian cancer by targeting TTK.
